# Knowledge and Concerns About Smoking‐Related Health Risks: A Cross‐Sectional Analysis of the 2021 International Tobacco Control Japan and Korea Surveys

**DOI:** 10.1111/dar.70043

**Published:** 2025-10-01

**Authors:** Tianze Sun, Gary Chan, Shannon Gravely, Anne C. K. Quah, Gang Meng, Geoffrey T. Fong, Steve S. Xu, Kota Katanoda, Hong Gwan Seo, Takahiro Tabuchi, Itsuro Yoshimi, Chang Bum Kang, Giang Vu, Ara Cho, Carmen Lim, Kayo Togawa, Sujin Lim, Sungkyu Lee, Sung‐il Cho, Gil‐yong Kim, Janni Leung

**Affiliations:** ^1^ National Centre for Youth Substance Use Research, School of Psychology The University of Queensland Brisbane Australia; ^2^ Department of Psychology University of Waterloo Waterloo Canada; ^3^ School of Public Health Sciences University of Waterloo Waterloo Canada; ^4^ Ontario Institute for Cancer Research Toronto Canada; ^5^ Division of Cancer Information Service Japan National Cancer Center Tokyo Japan; ^6^ Department of Disease Screening Design Hospital Jeonju Republic of Korea; ^7^ Division of Epidemiology, School of Public Health Tohoku University Graduate School of Medicine Sendai Japan; ^8^ National Institute of Infectious Diseases Tokyo Japan; ^9^ National Tobacco Control Center, Korean Health Promotion Institute Seoul Republic of Korea; ^10^ NHMRC Centre of Research Excellence on Achieving the Tobacco Endgame, School of Public Health, Faculty of Medicine The University of Queensland Brisbane Australia; ^11^ Early Detection, Prevention, and Infections Branch International Agency for Research on Cancer Lyon France; ^12^ Korea Center for Tobacco Control Research and Education Seoul Republic of Korea; ^13^ Graduate School of Public Health Seoul National University Seoul Republic of Korea

**Keywords:** attitudes, health literacy, knowledge, smoking, tobacco smoking, tobacco use

## Abstract

**Introduction:**

This cross‐sectional study examined: (i) knowledge of smoking‐related health risks among adults who currently and formerly smoke; (ii) concerns about personal health damage from smoking among adults who currently smoke; (iii) sociodemographic predictors of knowledge; and (iv) associations between knowledge and concerns in Japan and the Republic of Korea.

**Methods:**

Data from the 2021 International Tobacco Control Surveys included adults (aged ≥ 20, ≥ 19 respectively) in Japan (*n* = 2956 currently smoke, *n* = 852 formerly smoke) and Korea (*n* = 3776 currently smoke, *n* = 194 formerly smoke). Primary outcomes included knowledge of smoking‐related health risks (six consistently measured: stroke, heart disease, lung cancer, emphysema, impotence, early death), categorised as correct or incorrect, summed into a knowledge index score and concerns about smoking damaging their own health. Survey‐weighted analyses examined country and smoking status differences and associations between knowledge, concerns and sociodemographic characteristics.

**Results:**

Among adults who currently smoke, knowledge of lung cancer was highest (Japan: 82.8%, Korea: 92.2%); lowest were impotence in Japan (35.7%) and heart disease in Korea (69.6%). Adults who formerly smoked had higher knowledge than those who currently smoke in Japan (4.74 [4.50–4.97] vs. 4.00 [3.82–4.18]) but not in Korea (5.05 [4.39–5.72] vs. 4.69 [4.47–4.90]). Japanese adults who currently smoke had lower knowledge and fewer concerns than their Korean counterparts (*p* < 0.05). Greater knowledge predicted increased concerns in both countries (OR = 1.27 [1.20, 1.35]). Only in Japan were younger age (*B* = 0.59 [0.06, 1.13]) and moderate income (*B* = 0.41 [0.16, 0.66]) associated with greater knowledge.

**Discussion and Conclusions:**

The observed differences in knowledge and concerns about smoking‐related health risks between Japan and Korea may reflect their contrasting tobacco control policies.

## 1 Introduction

Tobacco smoking remains a leading cause of preventable death worldwide, contributing to over 8 million deaths annually, with 7 million from direct use and 1.3 million from second‐hand smoke exposure [1]. Smoking causes more than 52 diseases, including cardiovascular disease, chronic obstructive pulmonary disease and at least 16 types of cancer [1, 2]. Public awareness of smoking‐related health risks is essential for tobacco control efforts. The health behaviour change model posits that individuals' intentions to quit smoking are shaped by psychosocial factors, particularly one's knowledge of smoking‐related health harms and the degree of personal concern these risks evoke [3]. Individuals who understand the harms associated with smoking and feel personally concerned about these risks are more likely to attempt cessation. Therefore, identifying knowledge gaps and understanding how knowledge translates into concern is crucial for designing effective tobacco control policies.

Japan and the Republic of Korea (hereinafter referred to as Korea) share key structural features that make them ideal for comparative analysis within the International Tobacco Control (ITC) Asia‐Pacific Region. Both are high‐income East Asian countries with comparable healthcare systems, aging populations, a shared history of state‐owned tobacco companies (Korea has fully privatised its tobacco company while Japan has semi‐privatised Japan Tobacco) and historically high male smoking rates. While current smoking prevalence declined from 27.1% in 2011 to 19.3% in 2021 among Korean adults aged ≥ 19 [4] and from 20.1% in 2011 to 15.7% in 2023 among Japanese adults aged ≥ 20 [5], the burden of tobacco‐related disease remains substantial.

Japan and Korea have implemented distinct tobacco control policies (see Table S1, SI). Korea has adopted sustained public education campaigns about smoking risks [6, 7, 8] and introduced comprehensive advertising regulations. These include mandatory text and graphic health warnings covering 50% of the front and back of cigarette packages [9], bans on misleading terms such as ‘light’, ‘low tar’ and ‘mild’ [10], restrictions on advertising aimed at women and children, limits on magazine advertisements to 10 per year, and bans on promotional content that contradicts health warnings or implies health benefits [6]. In contrast, Japan relies on voluntary self‐regulation by the tobacco industry [11, 12, 13, 14], allows the use of misleading terms and is one of only two members of the Organisation for Economic Co‐operation and Development that still uses text‐only health warnings [15]. Japan has not implemented nationwide anti‐tobacco mass media campaigns and its limited messaging has historically focused on smoking etiquette, such as litter prevention, rather than health risks [14]. Despite these differences, both Japan and Korea fall short of fully adopting the six evidence‐based measures from the World Health Organization's MPOWER framework (Monitor tobacco use, Protect people from smoke, Offer cessation help, Warn about harms, Enforce advertising bans and Raise tobacco taxes) [16]. Other high‐income countries like Australia, Canada and the United Kingdom have implemented large graphic health warnings covering 75%–90% of cigarette packages, comprehensive smoke‐free laws, and complete bans on tobacco advertising, promotion and sponsorship.

Studies from nearly a decade ago show lower smoking‐related knowledge among adults who currently smoke and those with lower education or lower income in both countries [17, 18, 19]. However, these studies relied on non‐standardised measures, making cross‐country comparisons difficult and did not examine the relationship between knowledge and concern about personal health damage, which is a key precursor to behaviour change. This study addresses these gaps using data from the 2021 ITC Japan and Korea Surveys to examine:

1. knowledge of smoking‐related health risks (six consistently measured across countries: stroke, heart disease, lung cancer, emphysema, impotence and early death) among adults who currently and formerly smoke in Japan and Korea;
2. levels of concern about personal health damage from smoking among adults who currently smoke in Japan and Korea;
3. sociodemographic factors (age, sex, education, income) associated with knowledge scores among adults who currently smoke in Japan and Korea; and
4. the relationship between knowledge and health concerns among adults who currently smoke, accounting for sociodemographic factors.

## 2 Methods

This study follows the STROBE guidelines for reporting cross‐sectional studies [20]. Data were collected from Wave 4 of the ITC Japan Survey and Wave 2 of the ITC Korea Survey (both conducted in 2021). The study included adults (aged ≥ 20 in Japan and ≥ 19 in Korea) who either currently smoked cigarettes or had quit smoking cigarettes. Both surveys employed stratified, web‐based quota sampling through Rakuten Insight's online panel, with informed consent obtained prior to survey completion. In Japan, a recontact‐plus‐replenishment design was used to maintain the longitudinal cohort while recruiting new participants across four user‐type quotas (exclusive cigarette smokers, exclusive heated‐tobacco‐product users, dual users and non‐users), balanced by sex, age and region [21]. In Korea, participants were recruited across six user‐type strata (cigarette‐only, heated‐tobacco‐product‐only, e‐cigarette‐only, three dual‐use combinations and non‐users) to approximate census smoking distributions [22]. Both surveys used post‐stratification weights based on age × sex × region × user‐type to ensure samples represented the adult smoking populations in each country.

The original sample sizes were *N* = 4620 for Japan and *N* = 4683 for Korea. Participants who had never smoked (Japan, *n* = 653; Korea, *n* = 540) were removed from the analysis. We assessed missing data across all variables used in the analytic models, including smoking status, sociodemographic characteristics (sex, age, education, income), knowledge of smoking‐related health risks and concern about personal health damage from smoking (see Table S3, SI). Missing data for these variables ranged from 0.0% to 1.6%. Based on this, over 95% of participants had complete data, defined as having no missing values on any of the variables included in the analysis. Therefore, participants with missing data were excluded in a complete case analysis (Japan: *n* = 158; Korea: *n* = 173), resulting in final analytic samples of *N* = 3809 for Japan and *N* = 3970 for Korea.

### 2.1 Measures

#### 2.1.1 Smoking Status

Smoking status was determined using the question: ‘How often do you currently smoke cigarettes?’. In both surveys, cigarettes were explicitly defined to include only combustible tobacco cigarettes (factory‐made or hand‐rolled). Adults who currently smoke were defined as those who answered ‘Daily’, ‘Weekly’ or ‘Less than weekly’. Adults who formerly smoked (quitters) were defined as those who had reported having quit smoking completely at the time of the survey, regardless of how long ago they quit.

#### 2.1.2 Knowledge About Smoking‐Related Health Risks

Knowledge was assessed by asking participants to indicate whether smoking causes various health risks. Six health risks were measured consistently in both surveys, including five major smoking‐related diseases (stroke, heart disease, lung cancer, emphysema and impotence), as well as one mortality outcome associated with smoking (early death). Responses to each health risk were coded as: (1) correct [yes]; or (2) not correct [no/don't know]. Participants who refused to answer any question (Japan: *n* = 93; Korea: *n* = 158) were treated as missing.

A knowledge index was generated by summing the correct responses for six parallel health risks that were consistently measured across both countries. This score ranged from 0 to 6, with higher scores indicating greater knowledge of smoking‐related health effects. In addition to these six health effects, Japan's survey included eight additional effects, including heart attack, chronic obstructive pulmonary disease, asthma, bronchitis, low birth weight/premature birth, addiction/health damage, gastric ulcers, and damage to gums or teeth, while Korea's survey included three additional risks, including throat cancer, oral cancer and tooth discolouration. These country‐specific items provide supplementary insights into health risk awareness within each population but were not included in the knowledge index to maintain cross‐country comparability.

#### 2.1.3 Concern About Personal Health Damage From Smoking

Concern about smoking‐related health harms was only measured among adults who currently smoke, using a subjective item that asked participants how worried they were that smoking cigarettes would damage their future health. Responses were categorised as: (i) ‘Not at all’/‘A little’; (ii) ‘Moderately’/‘Very Worried’; (iii) ‘Don't know’. Participants who refused (Japan: *n* = 30; Korea: *n* = 14) were treated as missing (Table S3).

#### 2.1.4 Sociodemographic Characteristics

Sociodemographic characteristics included sex (male, female), age group (19/20–29, 30–39, 40–59 and ≥ 60), education level (Japan: low [junior high school, high school], moderate [vocational school, junior college/technical college], high [undergraduate or higher]; Korea: low [never attended school, dropped out of school, primary school or middle school], moderate [completed high school or some university without degree], high [completed university degree or postgraduate]) and income (Japan: low [≤ 4 million JPY], moderate [> 4 to 6 million JPY], high [> 6 million JPY]; Korea: low [< 30 million KRW], moderate [30 to < 75 million KRW], high [≥ 75 million KRW]).

### 2.2 Statistical Analysis

Analyses were conducted using Stata 17.0. Sample characteristics were described using frequencies and unweighted percentages, with Chi‐square tests comparing sociodemographic characteristics between Japan and Korea, stratified by smoking status to contextualise the study populations (Table 1). Subsequent analyses applied survey weights and strata using the *svyset* command to account for complex survey design and ensure population representativeness. Weights were calibrated to population benchmarks for smoking status based on the 2021 Japan Society and New Tobacco Internet Survey [23] and the 2020 Korean Community Health Survey [24].

**TABLE 1:** Participants' characteristics in the 2021 International Tobacco Control Japan and Korea Surveys, with chi‐square tests for between‐country differences among adults who currently smoke vs. those who formerly smoked.

Characteristics	Japan (*N =* 3809)				Korea (*N* = 3970)				Between countries	
	Currently smoke		Formerly smoked		Currently smoke		Formerly smoked		Currently smoke	Formerly smoked
	*N* = 2956		*N* = 853		*N* = 3776		*N* = 194		Chi‐square (*χ* ^2^), *p*	
	*n*	%	*n*	%	*n*	%	*n*	%		
Sex									*χ* ^2^ = 200.5, *p* < 0.05	*χ* ^2^ = 33.5, *p* < 0.05
Male	2149	72.7	471	55.2	3266	86.5	151	77.8		
Female	807	27.3	382	44.8	510	13.5	43	22.2		
Age group, years									*χ* ^2^ = 366.4, *p* < 0.05	*χ* ^2^ = 55.6, *p* < 0.05
19–29	92	3.1	25	2.9	438	11.6	21	10.8		
30–39	715	24.2	368	43.1	869	23.0	42	21.7		
40–59	1396	47.2	313	36.7	2048	54.2	106	54.6		
≥ 60	754	25.5	147	17.2	421	11.2	25	12.9		
Education									*χ* ^2^ = 1500, *p* < 0.05	*χ* ^2^ = 98.3, *p* < 0.05
Low	1005	34.0	279	32.7	29	0.8	2	1.0		
Moderate	571	19.3	186	21.8	689	18.3	35	18.0		
High	1380	46.7	388	45.5	3058	81.0	157	80.9		
Income									*χ* ^2^ = 1300, *p* < 0.05	*χ* ^2^ = 95.8, *p* < 0.05
Low	769	26.0	193	22.6	114	3.0	8	4.1		
Moderate	613	20.7	168	19.7	1922	50.9	94	48.5		
High	1239	41.9	398	46.7	1683	44.6	89	45.9		
Not reported	335	11.3	94	11.0	57	1.5	3	1.6		

*Note*: Unweighted numbers and proportions are presented. (a) Current smokers included those who smoked at least weekly; former smokers included those who smoked previously. Chi‐square (*χ* ^2^) and *p*‐values indicate statistically significant differences between Japan and Korea for each characteristic, within current and former smoker groups.

To examine knowledge levels (Aim 1), weighted proportions of correct responses were calculated for each smoking‐related health risk. Chi‐square tests compared total knowledge scores between adults who currently and formerly smoke within each country. For concern levels (Aim 2), weighted proportions were calculated only among adults who currently smoke, as concerns were not measured among adults who formerly smoked.

For demographic factors associated with knowledge (Aim 3), survey‐weighted linear regression analyses were conducted separately by country among adults who currently smoke, with sex, age, education and income as independent variables. Regression coefficients (*β*) and 95% confidence intervals were reported for each country to identify significant associations with knowledge scores. The regression coefficients represent the predicted changes in knowledge scores associated with each predictor variable while controlling for other variables in the model.

For knowledge‐concern associations among adults who currently smoke (Aim 4), survey‐weighted multinomial logistic regression was conducted using three models. Concern about personal smoking‐related health damage served as the outcome variable. Model 1 (unadjusted) included only knowledge score as a predictor. Model 2 (adjusted) added sociodemographic covariates: country, age group, education and income. Model 3 (fully adjusted) tested effect modification by adding an interaction term between knowledge score and country, adjusting for Model 2 covariates. The three‐category outcome (no/low concern, moderate/high concern, don't know) used no/low concern as the reference. Results are reported as odds ratios (OR) with 95% confidence intervals.

## 3 Results

### 3.1 Sample Characteristics

Table 1 presents the sociodemographic characteristics from the 2021 Japan and Korea surveys. Chi‐square tests indicated statistically significant differences between countries across all characteristics (*p* < 0.05). Among adults who currently smoke, both countries had higher proportions of males than females. Japan had more females than Korea (27.3% vs. 13.5%, *χ* ^2^ = 200.5, *p* < 0.05) and more adults aged ≥ 60 (25.5% vs. 11.2%, *χ* ^2^ = 366.4, *p* < 0.05). In Japan, 34.0% of adults who currently smoke had low education compared to only 0.8% in Korea (*χ* ^2^ = 1500, *p* < 0.05). Income distributions differed significantly, with more Japanese respondents in the low‐income category (26.0% vs. 3.0%, *χ* ^2^ = 1300, *p* < 0.05). Similar demographic patterns were observed among adults who formerly smoked. Overall country comparisons regardless of smoking status are presented in Table S4.

### 3.2 Knowledge of Smoking‐Related Health Risks

Table 2 presents weighted percentages for knowledge of smoking‐related health risks among adults who currently and formerly smoked in Japan and Korea in 2021.

**TABLE 2:** Weighted percentages and 95% confidence intervals for knowledge of smoking‐related health effects and concerns about health damage among adults who currently and formerly smoked in Japan and Korea (2021).

Knowledge of smoking related health effects	Weighted %s [95% confidence intervals]				
	Japan			Korea	
	Currently smoke (*N* = 2956)	Formerly smoked (*N* = 853)		Currently smoke (*N* = 3776)	Formerly smoked (*N* = 194)
Parallel measures (a)					
Stroke	71.3% [66.9%–75.3%]	80.9% [72.8%–87.0%]		74.2% [69.4%–78.5%]	90.4% [79.8%–95.7%]
Heart disease	67.5% [63.1%–71.6%]	83.5% [76.7%–88.6%]	*	69.6% [64.6%–74.2%]	73.1% [57.5%–84.5%]
Lung cancer	82.8% [79.5%–85.7%]	94.1% [89.7%–96.7%]	*	92.2% [90.2%–93.8%]	95.6% [83.1%–99.0%]
Emphysema	77.0% [72.8%–80.7%]	93.0% [88.4%–95.9%]	*	81.1% [76.4%–85.0%]	86.7% [66.8%–95.5%]
Impotence	35.7% [31.6%–40.1%]	37.1% [29.2%–45.8%]		74.0% [69.2%–78.4%]	75.2% [59.3%–86.3%]
Early death	66.0% [61.8%–70.0%]	85.3% [79.1%–89.9%]	*	77.5% [72.8%–81.6%]	81.4% [63.9%–91.6%]
Knowledge index (a)					
(Mean, SD)	4.00 [3.82–4.18]	4.74 [4.50–4.97]	*	4.69 [4.47–4.90]	5.02 [4.44–5.61]
Japan‐specific measures					
Heart attack	71.0% [67.1%–74.6%]	82.3% [75.2%–87.7%]		—	—
COPD	69.7% [65.4%–73.8%]	84.3% [77.4%–89.4%]	*	—	—
Asthma	74.0% [70.6%–77.2%]	89.0% [83.1%–93.0%]	*	—	—
Bronchitis	77.0% [73.6%–80.1%]	91.6% [86.5%–94.9%]	*	—	—
Low birth weight/premature	73.0% [69.2%–76.6%]	85.1% [77.9%–90.2%]	*	—	—
Addiction/health damage	75.7% [72.2%–78.9%]	89.4% [84.0%–93.1%]	*	—	—
Gastric ulcers	43.8% [39.5%–48.2%]	52.7% [44.0%–61.2%]		—	—
Damage to gums or teeth	60.3% [55.7%–64.7%]	72.6% [64.0%–79.7%]		—	—
Korea‐specific measures					
Throat cancer	—	—		88.1% [85.3%–90.4%]	85.6% [66.0%–94.8%]
Oral cancer	—	—		89.6% [87.3%–91.6%]	88.7% [66.9%–96.8%]
Tooth discolouration	—	—		92.3% [90.2%–93.9%]	95.3% [83.3%–98.8%]
Concern that smoking will damage health					
Not at all/A little	69.5% [64.6%–74.0%]	—		48.3% [42.5%–54.1%]	—
Moderately/very worried	19.2% [15.1%–24.2%]	—		46.5% [40.6%–52.5%]	—
Don't know	11.3% [8.7%–14.5%]	—		5.2% [4.1%–6.8%]	—

*Note*: Weighted estimates are presented. Currently, smoke included those who smoke daily, weekly, or less than weekly; Formerly smoked included those who quit for more than 30 days. (a) Knowledge index score out of 6, higher scores indicate greater knowledge of smoking‐related health effects, based on 6 health risks measured consistently in both Japan and Korea: stroke, heart disease, lung cancer, emphysema, impotence and early death.

Abbreviation: COPD, chronic obstructive pulmonary disease.

* Significant difference between smoking status (*p* < 0.05).

#### 3.2.1 Parallel Smoking‐Related Health Risks Measures

Among adults who currently smoke, lung cancer was most widely recognised in both countries (Japan: 82.8% [79.5%–85.7%]; Korea: 92.2% [90.2%–93.8%]), followed by emphysema (Japan: 77.0% [72.8%–80.7%]; Korea: 81.1% [76.4%–85.0%]). In Japan, the lowest recognised risks were impotence (35.7% [31.6%–40.1%]) and early death (66.0% [61.8%–70.0%]). In Korea, heart disease (69.6% [64.6%–74.2%]) and impotence (74.0% [69.2%–78.4%]) were the least recognised.

Similar patterns were observed among adults who formerly smoked. Japanese adults who formerly smoked showed significantly higher knowledge than those who currently smoke about heart disease, lung cancer, emphysema and early death (*p* < 0.05). In Korea, only stroke knowledge differed significantly, with adults who formerly smoked showing greater awareness (*p* < 0.05).

#### 3.2.2 Knowledge Index

Adults who formerly smoked demonstrated higher knowledge scores than those who currently smoke in both countries, though this difference was only statistically significant in Japan (4.74 [4.50–4.97] vs. 4.00 [3.82–4.18]) and not in Korea (5.02 [4.44–5.61] vs. 4.69 [4.47–4.90]). Korean adults who currently smoke had significantly higher knowledge scores (4.69 [4.47–4.90]) than Japanese adults (4.00 [3.82–4.18]). Among adults who formerly smoked, knowledge scores did not differ significantly between Korea (5.02 [4.44–5.61]) and Japan (4.74 [4.50–4.97]).

#### 3.2.3 Japan‐Specific Smoking‐Related Health Risks Measures

Among adults who currently smoke, bronchitis (77.0% [73.6%–80.1%]) and addiction/health damage (75.7% [72.2%–78.9%]) were the most recognised health risks. Less than half recognised that smoking caused gastric ulcers (43.8% [39.5%–48.2%]). Adults who formerly smoked showed similar patterns but demonstrated significantly higher knowledge than those who currently smoke for chronic obstructive pulmonary disease, asthma, bronchitis, low birth weight/premature birth, addiction/health damage and gastric ulcer risks (*p* < 0.05).

#### 3.2.4 Korea‐Specific Smoking‐Related Health Risks Measures

Among Korean adults who currently smoke, knowledge was high for tooth discolouration (92.3% [90.2%–93.9%]), oral cancer (89.6% [87.3%–91.6%]) and throat cancer risks (88.1% [85.3%–90.4%]). Adults who formerly smoked also showed high knowledge (> 85.6%), with non‐significant trends towards higher knowledge than adults who currently smoke.

### 3.3 Concerns About Personal Health Damage From Smoking Among Adults Who Currently Smoke

Table 2 shows weighted percentages for concern levels among adults who currently smoke. In Korea, nearly half (48.3% [42.5%–54.1%]) reported little or no concern, while 46.5% [40.6%–52.5%] reported being moderately or very worried about smoking damaging their health, and few (5.2% [4.1%–6.8%]) reported that they did not know if they were concerned. In Japan, 69.5% (64.6%–74.0%) expressed little or no concern. Only 19.2% [15.1%–24.2%] reported that they were moderately or very worried, and 11.3% [8.7%–14.5%] reported that they were uncertain.

When comparing the two countries, Korean adults who currently smoke reported substantially higher concern (46.5% [40.6%–52.5%]) than Japanese adults (19.2% [15.1%–24.2%]). Japan had significantly more adults reporting no/low concern (69.5% [64.6%–74.0%] vs. Korea: 48.3% [42.5%–54.1%]) and uncertainty about their concerns (11.3% [8.7%–14.5%] vs. 5.2% [4.1%–6.8%]). Concern was not measured among adults who formerly smoked in either country.

### 3.4 Sociodemographic Factors Associated With Knowledge Among Adults Who Currently Smoke

In Japan, age and income were significantly associated with knowledge scores (Table 3). Compared to those aged ≥ 60, higher knowledge scores were observed among adults aged 20–29 (*B* = 0.59 [0.06, 1.13]), 30–39 years (*B* = 0.52 [0.25, 0.78]) and 40–59 years (*B* = 0.35 [0.14, 0.56]), indicating a clear age gradient. Both moderate‐income (*B* = 0.41 [0.16, 0.66]) and high‐income groups (*B* = 0.25 [0.01, 0.48]) had higher knowledge scores than the low‐income group. Sex and education levels were not significantly associated with knowledge scores. In Korea, no sociodemographic factors were associated with knowledge.

**TABLE 3:** Linear regression analysis of factors associated with knowledge of smoking‐related health risks among adults who currently smoke in Japan and Korea (2021).

	Linear regression on knowledge score	
	Japan	Korea
	B [95% CI]	B [95% CI]
Sex (ref: female)		
Male	−0.09 [−0.29, 0.12]	−0.08 [−0.48, 0.32]
Age group, years (ref: ≥ 60)		
20–29	0.59 [0.06, 1.13]*	0.18 [−0.22, 0.57]
30–39	0.52 [0.25, 0.78]*	0.16 [−0.22, 0.54]
40–59	0.35 [0.14, 0.56]*	−0.02 [−0.36, 0.32]
Education (ref: high)		
Low	−0.09 [−0.30, 0.12]	−0.63 [−1.61, 0.35]
Moderate	0.20 [−0.05, 0.45]	−0.06 [−0.28, 0.15]
Income (ref: low)		
Moderate	0.41 [0.16, 0.66]*	0.29 [−0.30, 0.88]
High	0.25 [0.01, 0.48]*	0.30 [−0.32, 0.91]
Not reported	−0.26 [−0.58, 0.05]	0.07 [−0.89, 1.04]

*Note*: Analyses included responses from participants who were currently smoking in the Japan and Korea 2021 surveys.

Abbreviation: CI, confidence interval.

* *p* < 0.05.

### 3.5 Association Between Knowledge and Concern Among Adults Who Currently Smoke

Logistic regression revealed consistent associations between knowledge and health concerns (Table 4). In Model 1 (unadjusted), higher knowledge scores were associated with greater odds of moderate or high health concerns (OR = 1.31 [1.24, 1.39]) and lower odds of reporting uncertainty about concerns (OR = 0.71 [0.67, 0.77]).

**TABLE 4:** Multinomial logistic regression of the association between knowledge of smoking‐related harms and concerns that smoking will damage health among adults who currently smoke in Japan and Korea (2021).

	Multinomial logistic regression on concern levels in adults who currently smoke (ref: no/low)			
	Moderate/high		Don't know	
	OR [95% CI]		OR [95% CI]	
Model 1: unadjusted				
Knowledge	1.31 [1.24, 1.39]	*	0.71 [0.67, 0.77]	*
Model 2: with covariates				
Knowledge	1.27 [1.20, 1.35]	*	0.71 [0.67, 0.77]	*
Country (ref: Korea)				
Japan	0.26 [0.21, 0.33]	*	0.65 [0.45, 0.93]	*
Sex (ref: female)				
Male	0.92 [0.72, 1.19]		0.73 [0.51, 1.05]	
Age group (ref: ≥ 60)				
20–29	0.62 [0.42, 0.91]	*	0.49 [0.25, 0.94]	*
30–39	0.89 [0.65, 1.22]		0.76 [0.50, 1.16]	
40–59	0.99 [0.76, 1.29]		0.84 [0.59, 1.19]	
Education (ref: high)				
Low	1.17 [0.86, 1.59]		1.34 [0.96, 1.89]	
Moderate	1.07 [0.87, 1.32]		1.69 [1.23, 2.32]	*
Income (ref: low)				
Moderate	0.98 [0.70, 1.38]		0.57 [0.35, 0.92]	*
High	1.10 [0.79, 1.52]		0.68 [0.45, 1.03]	
Not reported	0.72 [0.44, 1.17]		2.14 [1.37, 3.35]	*
Model 3: with interaction				
Knowledge × country	0.96 [0.86, 1.08]		0.98 [0.85, 1.14]	

*Note*: Analyses included responses from adults who currently smoke in the Japan and South Korea 2021 surveys. Model 3 was conducted adjusting for all covariates in model 2.

Abbreviations: CI, confidence interval; OR, odds ratio.

* *p* < 0.05.

These associations persisted after adjusting for sociodemographic factors (Model 2). Higher knowledge scores were associated with moderate/high concern (OR = 1.27 [1.20, 1.35]). Japanese participants had substantially lower odds of both moderate/high concerns (OR = 0.26 [0.21, 0.33]) and uncertainty compared to Korean participants (OR = 0.65 [0.45, 0.93]). Adults aged 20–29 years showed lower odds of moderate/high concern (OR = 0.62 [0.42, 0.91]) and uncertainty about concerns than those aged ≥ 60 (OR = 0.49 [0.25, 0.94]).

Education and income were not significantly associated with moderate/high concern, though moderate education (OR = 1.69 [1.23, 2.32]) and unreported income (OR = 2.14 [1.37, 3.35]) were associated with increasing the odds of uncertainty about concerns. Model 3 found no significant interaction between knowledge and country, indicating consistent knowledge–concern relationships across countries.

## 4 Discussion

Our findings revealed differences in knowledge of smoking‐related health risks between Japan and Korea, with Korean adults who currently smoke demonstrating higher overall knowledge and health concerns than their Japanese counterparts. Higher knowledge was consistently associated with greater concerns about personal health damage from smoking in both countries, even after adjusting for sociodemographic factors.

Health behaviour change theories and the ITC conceptual model suggest that policies (e.g., graphic warning labels) can influence one's knowledge and concerns about smoking‐related harms, which ultimately influence outcomes that are important in public health (e.g., quitting behaviour) [3]. Although we did not directly measure individual exposure to tobacco control policies, the observed differences in knowledge and concerns between Japan and Korea may reflect their contrasting tobacco control environments. The higher knowledge and concern levels observed in the Korean participants may result from comprehensive tobacco control measures, including graphic health warnings [9], advertising bans [6] and public education campaigns [7]. In contrast, Japan's tobacco control measures remain relatively limited, relying on text‐only warnings [15], continued use of misleading descriptors on cigarette packaging, and a lack of comprehensive bans on tobacco advertising, which are policies proven less effective at increasing smoking‐related knowledge or motivations to quit smoking [8, 13, 15, 25].

Participants who reported currently smoking in either country recognised that smoking causes lung cancer and emphysema. However, awareness varied for other risks. In Japan, knowledge was lowest for impotence (35.7%) and early death (66.0%), while in Korea, heart disease (69.6%) and impotence (74.0%) were the least recognised. The lower awareness of impotence risks observed among the Japanese sample may be due to its exclusion from cigarette package warnings and public education campaigns, in contrast to Korea, where impotence appears explicitly on cigarette packaging and in public health messaging [9].

Consistent with behavioural change theories and prior research [18, 26], adults who formerly smoked had higher knowledge scores than adults currently smoking in both countries, though the difference was only statistically significant in the Japanese sample. The lack of significance in the Korean sample could be attributed to a ceiling effect, where baseline knowledge is already relatively high, potentially due to stronger tobacco control measures. Alternatively, unmeasured psychosocial factors, such as social norms, self‐efficacy, or cognitive dissonance, may play a role in smoking behaviour [3]. Under Cognitive Dissonance Theory, individuals experience psychological conflict between their knowledge of smoking‐related risks and their choice to continue smoking. To manage this discomfort, participants may adopt dissonance‐reducing beliefs that downplay risks or emphasise perceived benefits, but such beliefs reduce once they quit smoking [27].

Consistent with previous research on widening socioeconomic inequalities in smoking prevalence among lower‐income groups in Japan [28], younger and higher‐income individuals who currently smoke demonstrated greater knowledge in our study. Reducing these knowledge gaps may require strengthening tobacco control policies targeting lower‐income and older populations, expanding education campaigns and improving smoking cessation service accessibility.

### 4.1 Limitations and Future Research

Several limitations should be noted. First, the impact of individual policies implemented in each country cannot be directly linked to knowledge and concern. Longitudinal studies measuring policy exposure (e.g., tobacco advertising, anti‐tobacco campaigns, health warnings) could examine how knowledge and concerns change in response to specific tobacco control initiatives over time. Second, self‐reported concerns about smoking‐related harms may reflect individual health perceptions, anxiety levels, or social desirability bias, though standardised questions ensured consistency across participants. Third, assessing knowledge through close‐ended questions about ‘true’ health effects may cause overestimation through agreement bias. Lastly, while our focus was on knowledge of smoking‐related health risks among individuals who currently and formerly smoke, regardless of other product use, the categorisation of individuals who currently and formerly smoke may mask important differences in nicotine product use patterns. Previous ITC studies in Japan [29] and Korea [30] show that many individuals who formerly smoked cigarettes now use alternative nicotine products like heated tobacco products [31] rather than achieving complete nicotine cessation.

Future research should examine knowledge variations across different patterns of nicotine product use and investigate how social norms, cultural attitudes, and tobacco industry influence shape smoking‐related knowledge, concerns and behaviours in both countries.

### 4.2 Implications

Effective communication of smoking‐related health risks is a primary goal of tobacco control policy. Governments and tobacco companies should be responsible for communicating these risks to the public, particularly lesser‐known risks. Evidence‐based approaches like graphic health warnings on cigarette packages can reach diverse populations effectively because they provide easy access to health information across demographics, and potentially reduce knowledge disparities among older and lower‐income adults [25].

## Author Contributions

Data collection: Geoffrey T. Fong, Anne C.K. Quah, Steve S. Xu, Kota Katanoda, Takahiro Tabuchi, Itsuro Yoshimi, Sungkyu Lee, Chang Bum Kang, Gil‐yong Kim, Sujin Lim, Hong Gwan Seo and Sung‐il Cho. Design and concept: Janni Leung, Tianze Sun, Gary Chan, Geoffrey T. Fong, Gang Meng, Shannon Gravely, Anne C.K. Quah and Steve S. Xu. Data analysis: Tianze Sun, Janni Leung and Gang Meng. First draft: Janni Leung, Ara Cho, Giang Vu and Tianze Sun. Subsequent drafts: all authors. Final version and accountability: all authors.

## Disclosure

Where authors are identified as personnel of the International Agency for Research on Cancer/World Health Organization, the authors alone are responsible for the views expressed in this article and they do not necessarily represent the decisions, policy or views of the International Agency for Research on Cancer/World Health Organization.

## Ethics Statement

The ITC Japan survey protocols and all materials, including the survey questionnaires, were cleared for ethics by the Research Ethics Board, University of Waterloo, Canada (REB#22508/31428), the Internal Review Board at the Osaka International Cancer Institute, Japan (IRB 21054), and the Internal Review Board at Japan National Cancer Center, Japan (IRB 2021‐069). The ITC Korea Survey was reviewed and cleared by the Research Ethics Board, Office of Research Ethics at the University of Waterloo, Canada (REB#41512), and the Institutional Review Board, Korea Health Promotion Institute, Republic of Korea (#120160811107AN01‐2004‐HR‐042‐02).

## Consent

All participants provided consent to participate.

## Conflicts of Interest

G.T.F. has served as an expert witness or consultant for governments defending their country's policies or regulations in litigation. All other authors declare no conflicts of interest. The funders had no role in the study's design, in the collection, analysis, or interpretation of data, the writing of the manuscript, or the decision to publish the results.

## Supporting information


**Data S1:** Supporting Information.

## Data Availability

In each country participating in the international Tobacco Control Policy Evaluation (ITC) Project, the data are jointly owned by the lead researcher(s) in that country and the ITC Project at the University of Waterloo. Data from the ITC Project are available to approved researchers 2 years after the date of issuance of cleaned data sets by the ITC Data Management Centre. Researchers interested in using ITC data are required to apply for approval by submitting an International Tobacco Control Data Repository request application and subsequently to sign an International Tobacco Control Data Repository Data Usage Agreement. The criteria for data usage approval and the contents of the Data Usage Agreement are described online (http://www.itcproject.org).
