# Supplementary material for: Knowledge and Concerns About Smoking‐Related Health Risks: A Cross‐Sectional Analysis of the 2021 International Tobacco Control Japan and Korea Surveys
Source: Drug Alcohol Rev. 2025 Oct 1;44(7):2127–37. doi: 10.1111/dar.70043 (PMC12581927; doi:10.1111/dar.70043)
Supplement: Supplementary file 1 — Data S1: Supporting Information. [file DAR-44-2127-s001.docx]

# **Supporting Information**

# *Table S1. Cigarette-related policies in Japan and Korea.*

| **Domain (Article number in World Health Organization Framework Convention on Tobacco Control)** | | **Japan** | **Korea** |
| --- | --- | --- | --- |
| Product controls | Product regulation (Art 9) | Mandatory display of nic and tar on packs; domestic tob production only by Japan Tobacco; Importation & sale allowed for other tob companies with MOF permits | ‘Tobacco’ definition expanded to any part of tob plants; nic level limited to 1% (>1% requires intensive approval process; making >1% onerous) |
|  |  |  |  |
|  | Flavours (Art 9) | No restrictions | No restrictions |
| Price controls | Taxes (Art 6) | Total specific excise tax in 2023: ¥304.88 (PPP$3.12)/20 cigs (50.8% of retail price); VAT 10% | Total specific excise tax in 2023: ₩2913 (PPP$3.50)/20 cig sticks (64.8% of retail price); VAT 10% |
| Place controls | Availability & Access | Available in convenience & grocery stores | Available in convenience & grocery stores |
|  | Smoke-free  (Art 8) | Ban in certain public indoor places; designated smoking rooms allowed; small venues (<100m^2^) exempted | Ban in certain public indoor places; nightclubs allowed; small venues (<1000m^2^) exempted |
| Promotional controls | Advertising & Promotion  (Art 13) | Not prohibited; MOF guidance on ad & promo via voluntary ‘guidance by industry (self-regulation)’: Tob ads restricted on billboards, buildings, transportation, but allowed at POS, smoking places, & places for adult venues | Ads prohibited on tv & radio; print ads in magazines allowed except those targeted at women & children; print ads allowed ≤10 times/yr & must not encourage/induce non-cigarette consumers to smoke, or include contents contradicting smoking warnings |
|  |  |  |  |
|  | Health Warnings  (Art 11) | THWs; 50% front & back; 5 THWs (e.g., diseases, harms to self & others); misleading descriptors (‘low tar’, ‘light’, ‘mild’) allowed, with disclaimers | 50% PHW front & back (e.g., diseases, harms to self & others, tar, carcinogens, phone #s for smoking cessation); 10 PHWs; misleading descriptors (‘light’, ‘low tar’, ‘genuine’) banned |
| Future Policies | | Health Promotion Act 2020 may be reviewed and amended in 2025 Based on best available reports + consultation with ITC Japan experts: • Tax (Art 6): increase ¥3 (US$0.02) per tob (cig & HTP) stick in stages in/after 2024; • Smoke-free (Art 8): no changes are likely  • Warnings (Art 11): possible enhancements but will stay text-only • Advertising & promotion (Art 13): no changes are likely • Cessation (Art 14): no changes are likely | 5th National Health Plan 2021-2030 (HP2030): • Product Regulation (Art 9): regulates all nic products (ECs, HTPs) as tob products, including those with synthetic nic; may ban/restrict flavors in all products • Tax/price (Art 6): likely to increase taxes on all products • Smoke-free (Art 8): will strengthen existing laws—100% smoke-free and nic-free in all indoor buildings • Warnings (Art 11): likely to increase PHWs to 75% and introduce plain packaging • Advertising & promotion (Art 13): likely to strengthen existing laws • Cessation (Art 14): will reinforce prevention programs, customize cessation services for high-risk & vulnerable pops, expand smoking prevention education, & consolidate all smoking cessation services |

HTP, heated-tobacco-product

#### Table S2. Tobacco-related knowledge variables measured across the 2021 International Tobacco Control Japan and Korea Surveys.

| **Knowledge items – Smoking causes:** | **Japan 2021** | **Korea 2021** |
| --- | --- | --- |
| Stroke | x | x |
| Heart disease | x | x |
| Heart attack | x | – |
| Lung cancer | x | x |
| Emphysema | x | x |
| COPD | x | – |
| Asthma | x | – |
| Bronchitis | x | – |
| Throat cancer | – | x |
| Oral cancer | – | x |
| Low birthweight or premature delivery from smoking during pregnancy | x | – |
| Addiction and/or health damage to underage smokers | x | – |
| Gastric ulcers | x | – |
| Damage to gums or teeth | x | – |
| Tooth discolouration | – | x |
| Impotence | x | x |
| Early death | x | x |

*Note:* x: measure available in survey; –: measure not available in survey.

COPD, chronic obstructive pulmonary disease.

#### Table S3. Level of missing data in the 2021 International Tobacco Control Japan and Korea Surveys

|  | **Japan (N = 3809)** | | | | **Korea (N = 3970)** | | | |
| --- | --- | --- | --- | --- | --- | --- | --- | --- |
|  | **Currently smoking** | | **Formerly smoking** | | **Currently smoking** | | **Formerly smoking** | |
|  | *N* = 3094 | | *N* = 873 | | *N* = 3946 | | *N* = 197 | |
|  | n | % | n | % | n | % | n | % |
| **Variables** |  |  |  |  |  |  |  |  |
| Sex | 0 | 0.0% | 0 | 0.0% | 0 | 0.0% | 0 | 0.0% |
| Age group | 0 | 0.0% | 0 | 0.0% | 0 | 0.0% | 0 | 0.0% |
| Education | 51 | 1.6% | 9 | 1.0% | 15 | 0.4% | 1 | 0.5% |
| Income | 0 | 0.0% | 0 | 0.0% | 0 | 0.0% | 0 | 0.0% |
| Current smoking status | 0 | 0.0% | 0 | 0.0% | 0 | 0.0% | 0 | 0.0% |
| **Knowledge that smoking causes:** |  |  |  |  |  |  |  |  |
| Stroke | 26 | 0.8% | 0 | 0.0% | 38 | 1.0% | 1 | 0.5% |
| Heart disease | 23 | 0.7% | 3 | 0.3% | 47 | 1.2% | 0 | 0.0% |
| Heart attack | 27 | 0.9% | 2 | 0.2% | -- | -- | -- | -- |
| Lung cancer | 26 | 0.8% | 2 | 0.2% | 37 | 0.9% | 0 | 0.0% |
| Emphysema | 29 | 0.9% | 3 | 0.3% | 47 | 1.2% | 0 | 0.0% |
| COPD | 24 | 0.8% | 5 | 0.6% | – | – | – | – |
| Asthma | 23 | 0.7% | 4 | 0.5% | – | – | – | – |
| Bronchitis | 29 | 0.9% | 5 | 0.6% | – | – | – | – |
| Throat cancer | – | – | – | – | 40 | 1.0% | 1 | 0.5% |
| Oral cancer | – | – | – | – | 33 | 0.8% | 1 | 0.5% |
| Low birthweight/premature delivery | 25 | 0.8% | 4 | 0.5% | – | – | – | – |
| Addiction or health damage | 26 | 0.8% | 6 | 0.7% | – | – | – | – |
| gestic ulcers | 24 | 0.8% | 5 | 0.6% | – | – | – | – |
| Damage to gums or teeth | 25 | 0.8% | 5 | 0.6% | – | – | – | – |
| Tooth discolouration | – | – | – | – | 46 | 1.2% | 0 | 0.0% |
| Impotence | 26 | 0.8% | 3 | 0.3% | 54 | 1.4% | 0 | 0.0% |
| Early death | 27 | 0.9% | 4 | 0.5% | 48 | 1.2% | 0 | 0.0% |
| **Concern that smoking will damage health** | 30 | 1.0% | – | – | 14 | 0.4% | – | – |
| **Across all variables** |  |  |  |  |  |  |  |  |
| Any missing data | 122 | 3.9% | 20 | 2.3% | 160 | 4.1% | 3 | 0.2% |
| Complete data | 2972 | 96.1% | 853 | 97.7% | 3786 | 95.9% | 194 | 98.5% |

*Note.* – not measured in survey.

COPD, chronic obstructive pulmonary disease.

*Table S4. Sociodemographic characteristics of all participants in the 2021 International Tobacco Control Japan and Korea Surveys, with chi-square tests for differences between countries.*

|  |  | Japan (*N=*3809) | | Korea (*N*=3970) | | Between countries | |
| --- | --- | --- | --- | --- | --- | --- | --- |
|  |  | *n* | % | *n* | % | Chi-square (χ²) | P-values |
| Gender |  |  |  |  |  | χ² = 334.2324 | p = 0.000 |
|  | Male | 2620 | 68.8 | 3417 | 86.1 |  |  |
|  | Female | 1189 | 31.2 | 553 | 13.9 |  |  |
| Age group |  |  |  |  |  | χ² = 421.5609 | p = 0.000 |
|  | 19-29 | 117 | 3.1 | 459 | 11.6 |  |  |
|  | 30-39 | 1083 | 28.4 | 911 | 23.0 |  |  |
|  | 40-59 | 1706 | 44.8 | 2154 | 54.3 |  |  |
|  | ≥60 | 903 | 23.7 | 446 | 11.2 |  |  |
| Education |  |  |  |  |  | χ² = 1600 | p = 0.000 |
|  | Low | 1284 | 33.7 | 31 | 0.8 |  |  |
|  | Moderate | 757 | 19.9 | 724 | 18.2 |  |  |
|  | High | 1768 | 46.4 | 3215 | 81.0 |  |  |
| Income |  |  |  |  |  | χ² = 1500 | p = 0.000 |
|  | Low | 962 | 25.3 | 122 | 3.1 |  |  |
|  | Moderate | 781 | 20.5 | 2016 | 50.8 |  |  |
|  | High | 1637 | 43.0 | 1772 | 44.6 |  |  |
|  | Not reported | 429 | 11.3 | 60 | 1.5 |  |  |

*Note.* Unweighted numbers and proportion are presented. Chi-square (χ²) and p-values indicate statistical significance of differences between Japan and Korea for each sociodemographic variable. Both Japan and Korea include participants who currently and formerly smoked.
